# Home Information and Communication Technology Use and Student Academic Performance: Encouraging Results for Uncertain Times

**DOI:** 10.3389/fpsyg.2021.638319

**Published:** 2021-06-23

**Authors:** David Robert Skvarc, Matthew Talbot, Travis Harries, Christopher J. Wilson, Nicki Joshua, Linda K. Byrne

**Affiliations:** ^1^Deakin University, Geelong, VIC, Australia; ^2^Pearson Clinical Assessment, Melbourne, VIC, Australia; ^3^Melbourne School of Psychological Sciences, The University of Melbourne, Parkville, VIC, Australia

**Keywords:** academic performance, information technology, home learning, technology access, home education

## Abstract

This study set out to examine the associations of certain information communication technology (ICT) factors in the home environment with academic performance. We employed existing data sets collated by Pearson Clinical Assessment in 2016 which included the WIAT-III A&NZ (Wechsler Individual Achievement Test - Australian and New Zealand Standardised, Third Edition) completed by 714 students aged between 4 and 18 years old, and the home environment questionnaire (HEQ) completed by the parents of those children. Sequential multiple regression models were used to analyze the complex interactions between home ICT factors and measures of student reading, writing, mathematical, and oral ability. The findings of this study indicate that after accounting for the known powerful predictors of household income and parental education: (a) a student’s access to an ICT rich home environment, (b) their aptitude in using home ICT, and (c) their recreational use of home ICT, are largely unrelated to academic performance. We observed some small positive correlations between academic performance and child ICT affinity, but also comparably sized negative associations with use of social media and educational TV viewing. Encouragingly, we propose that these findings suggest that increasing levels of ICT use and access in the home are unlikely to be detrimental to academic progress. These results provide important information for parents and educators given the impact of the Coronavirus global pandemic and the near world-wide adoption of ICT for home-schooling.

## Introduction

The ongoing, global digital revolution of the early 21st Century has been characterized by the convergence of technology and human development, where traditional student learning has adapted to the new digital environment (Scherer et al., 2019). As a result of this convergence, ‘e-learning,’ the use of information and communications technology (ICT) for learning at home and in the classroom, has found a prominent role in modern education systems (Genlott and Grönlund, 2016). This growing prevalence of ICT has been evident over the past 20 years in Australia. Household access to ICT has become ubiquitous; 97% of families with children under the age of 15 are connected to the internet and own an average of 7.8 connected devices (Australian Bureau of Statistics, 2018). In Australia, this uptake of home ICT and e-learning has invoked education policy, pedagogy, and curriculum changes. For instance, ICT literacy was introduced as a measure in the National Assessment Program - Literacy and Numeracy (NAPLAN) tests in 2005 (Thomson, 2015). Further, the Rudd Government’s Digital Education Revolution program in 2008 aimed to achieve a 1:1 computer to student ratio (Digital Education Revolution [DER], 2013). ICT has recently been included as a general life capability in the National Curriculum (Australian Curriculum, Assessment and Reporting Authority [ACARA], 2019).

The intentional approach to achieve greater student access to technology has been driven by the belief that technology access can facilitate improved academic outcomes. Such an intentional approach is consistent with Bronfenbrenner’s ecological systems model whereby the individual is developed by (and in turn, reciprocates the development of) their environmental contexts (Bronfenbrenner and Morris, 1998). These contexts range from the proximal such as the family and home environments, to the more distal settings of schools, communities, and governmental jurisdictions (Bronfenbrenner and Morris, 2006). Within the home, the transactional dyad between home and school has been described as the Home Learning Environment (HLE), where child access and interaction with educational resources at home predicts academic outcomes (for example, Lehrl et al., 2021). Research has generally found support for a significant correlation between HLE and longitudinal academic outcomes, even where enhancements to the HLE are domain incongruent. For example, provision of additional home reading materials and informal literacy activities at home was associated not just with improved literacy but also numeracy skills (Dimosthenous et al., 2020; Lehrl et al., 2020). Further, comparable associations have been observed between home ICT access and improved academic performance (Attewell et al., 2003; Fiorini, 2010; Casey et al., 2012; Lee and Wu, 2012; Lei and Zhou, 2012; Erdogdu and Erdogdu, 2015; Genlott and Grönlund, 2016). However, while these results have been used to promote programs aimed at increasing access in areas of low ICT penetration, positive findings have been far from universal with recent null findings (Fairlie and Robinson, 2013; Talaee et al., 2019) and others demonstrating a negative relationship between ICT access and academic outcomes (Vigdor et al., 2014; Hu et al., 2018). These mixed findings suggest there may be other influential factors as access alone may not, in all cases, enhance a student’s ability to learn.

In an attempt to reconcile findings in the field and account for numerous confounds, Skryabin et al. (2015) analyzed data from large scale international databases collected from 43 countries to investigate how a student’s use of home ICT influenced their achievement in reading, mathematics, and science. Household resources moderate several crucial predictors of academic performance, such as greater parental involvement, household educational attainment, and child access to classrooms with fewer students (Tucker-Drob and Bates, 2016; Barger et al., 2019). As such, socioeconomic status (SES) has routinely been observed as a reliable predictor of academic achievement, and the financial burden of ICT devices suggests that the academic influence of the two factors would be difficult to disentangle. Skryabin et al. (2015)’s findings showed that after accounting for SES and gender, frequency of home ICT usage for school-related tasks positively affected a student’s reading and mathematics achievement. Interestingly, the study also found that using home ICT, even for entertainment purposes, was positively associated with a student’s reading ability.

However, additional research investigating the extent to which home ICT factors influence academic outcomes is necessary. One crucial aspect of a student’s home technology use is the overall aptitude for ICT. Despite a general moral panic over increasing technology use by children, [see, for example, Koumachi (2019)] qualitative research shows that Australian parents largely value the assistance of ICT in their children’s learning (Plowman et al., 2010). For example, Hatzigianni and Margetts (2014) suggest that Australian families consider a child’s ICT literacy development in the home as significantly important for their future educational and employment success. In combination, these findings suggest that despite some reservations, families accept and support the increased use of ICT in the home for education purposes.

Unal and Unal (2017) claim that the success of e-learning could be attributed to successful fostering of facilitation of student communication, collaboration, and positive learning attitudes. Theories of pedagogy suggest that e-learning platforms enhance a student’s academic performance, in that the mechanics of the e-learning encourage higher levels of self-directed and autonomous learning than would be associated with analog learning styles (Kozikoğlu, 2019). For example, self-directed and autonomous learning fosters individual responsibility for identifying needs and resources, forming goals, implementing strategies and evaluating outcomes (Kozikoğlu, 2019). These skills have a positive correlation with higher academic performance (Jaleel and OM, 2017). Further, effective use of ICT empowers students to control their learning and encourages cognitive processes that are beneficial to developing learning skills, such as problem-solving, critical thinking, communication, and self-efficacy (Lee and Wu, 2012). Genlott and Grönlund (2016) measured these effects in primary students by comparing academic outcomes of students with varying degrees of access to an e-learning platform and observed that those with increasing access to the platform demonstrated stronger academic outcomes, and those who used the collaborative features demonstrated this effect even more prominently. This reflects Lee and Wu’s findings (2012) that the mechanics of e-learning are intrinsically effective to enhance learning skills, and even more so when ICT is intentionally used to foster collaboration.

Given the capacity for learning that is intrinsic in the mechanics of ICT use, the effect of a child’s general interactions with technology at home, outside of the context of formal learning, has also become an important focus of research. Using a large mixed-methods study employing in-depth qualitative analysis, Furlong and Davies (2012) present strong support for ICT use benefits in the home environment. Furlong and Davies theorize that as a result of ICT saturation, learning is no longer limited to traditional classifications of formal and informal learning, that is, schoolwork and homework, but also quasi-formal and incidental learning, such as through relaxation and play. This suggests that children are continually learning through their everyday interactions with technology, rather than solely through structured and focused tasks at school as a result of integrating digital information in daily home life. This finding of continual digital learning has been corroborated by Skryabin et al.’s (2015). Furlong and Davies (2012) describe this phenomenon as a decreasing distinction between school and home, and further suggest that the home is the primary site for developing ICT literacy skills. This implies that home ICT usage, including recreational usage such as social networking and web-surfing, may positively affect a child’s overall learning ability (Wittwer and Senkbeil, 2008; Lee and Wu, 2013).

The importance of the context and interactions of a child’s home ICT use is commonly a missing or peripheral factor in previous studies. Wittwer and Senkbeil’s (2008) assessment of 4,660 German students demonstrates the complexity of the relationship, finding that neither access to, nor usage of a computer, influenced a student’s mathematics test results. However, students who used computers in a self-determined way displayed stronger problem-solving skills, supporting the hypothesis that increased aptitude for ICT use may be a moderating factor for overall academic improvement. By contrast, the large international study by Hu et al. (2018) found that home ICT for school use was associated with poorer performance in literacy, mathematics, reading and science, leading the authors to speculate that home ICT provides a source of distraction in the forms of social and digital media. While other studies have supported these results [see, for instance, Bavelier et al. (2010) and Comi et al. (2017)], these results do not account for the full context of e-learning as outlined by Unal and Unal (2017), in which the home ICT environment is a proxy of informal learning. This was evident as secondary findings in this same study showed that recreational ICT use was associated with better performance in reading and science indices (Hu et al., 2018). Further analysis showed that higher interest in general ICT, competence in ICT, and autonomy in using ICT were all significantly associated with higher academic performance. While these authors did not actively promote home ICT as a positive influence of formal learning, their evidence still supports the argument of this research; that engagement with home ICT has a positive effect on academic performance, given that the students engage with formal and recreational technology platforms in ways conducive to e-learning.

The current study aims to examine and clarify the associations of home ICT access, aptitude, e-learning, and ICT recreation with student academic performance. We hypothesize that student access to home ICT will be positively correlated with higher academic performance (H1). Secondly, we aim broaden the scope to consider ICT literacy in the home and hypothesize that student ICT aptitude will be positively associated with higher academic performance (H2). Lastly, this study will consider the associations of different forms of e-learning, hypothesizing that student frequency of structured e-learning in the home will be positively associated with higher academic performance (H3), and student frequency in recreational ICT use (non-formal e-learning) will be positively correlated with academic performance (H4).

## Materials and Methods

### Data Sources, Instruments and Measures

#### Wechsler Individual Achievement Test III – Australian and New Zealand Standardization Sample

One of the main data sources of this research was the WIAT-III A&NZ, Wechsler Individual Achievement Test - Australian and New Zealand standardization sample, stratified to ensure that the normative sample included representative proportions of children matched to census data according to selected demographic variables, including geographical region, parental education, and age (Pearson, 2016). The WIAT-III A&NZ provides a standardized measure of overall academic performance and identification of academic strengths and weaknesses through subtest measures that can then be used for targeted education and learning interventions. In this case, the research will examine scores on the WIAT-III A&NZ academic indices: Reading, Writing, Oral and Mathematics scores. The scores on these indices have been scaled for age around a mean score of 100 and standard deviation of 15, allowing comparison across participants. The original data set contains scores from *n* = 1360 students, aged 4–19:11 and census-matched, who completed either the WISC-V or the WPPSI-IV dependent upon age. The WIAT-III A&NZ was administered to a total of 741 students as an individual performance-based assessment by a qualified psychologist. Exclusionary criteria were used to preclude some participants from the normative sample group. These criteria included (but was not limited to) the following:

• Primary language was not English.
• Uncorrected visual impairment or hearing loss.
• Abnormal fine or gross motor ability.
• Non-verbal or uncommunicative.
• Taking medication which might affect test performance.
• Diagnosis of a condition or illness which might affect test performance, such as a learning disability.
• Identical sibling to another participant in the study.

The participant sample was stratified to ensure that the normative sample included representative proportions of children matched to census data according to selected demographic variables, including geographical region, parental education, and age.

Parents were also requested to complete an optional Home Environment Questionnaire (HEQ). The age range of participants whose parents completed the HEQ was between 4–19:11 years of age (*M* = 11.13, *SD* = 3.11). Of the original 1,360 participants, *n* = 619 did not complete the HEQ. Seven-hundred and forty-one participants completed both the WIAT-III A&NZ and the HEQ. All duplicate records were excluded from this study. After these exclusions (*n* = 27), a total of *n* = 714student records were included in the final analysis. Analysis revealed that missing data were MCAR [χ^2^(722) = 855, *p* < 0.001], and pattern analysis suggested monotonicity. As such, multiple imputation (25 imputations) was used to estimate the missing values in the analyses.

#### Home Environment Questionnaire

This study’s second data source was a modified Home Environment Questionnaire (HEQ), an internally developed set of questions used by Pearson education to locate and test representative samples. The HEQ is a self-reporting instrument used to identify an array of psychosocial factors present in a child’s home environment. Pearson Clinical researchers first developed the HEQ to develop and standardize the WPPSI-IV US in 2010–2012. Since then, the HEQ has been used in the development and standardization of the WPPSI-IV A&NZ, WISC-V A&NZ, WIAT-III A&NZ, Bayley-4 A&NZ, and CELF-P3 A&NZ; and the internal scales of the HEQ have been used to predict academic risk with adequate reliability and validity (Kaufman et al., 2015). For this study, the modified HEQ provided both demographic information and data related to the child’s use of technology. Our modifications involved some selection of items based on our analyses for appropriate predictors. To apply this data in sequential multiple regression, each of the relevant measures were re-coded from a categorical to a numeric scale. The relevant items and Cronbach’s alpha (where appropriate) are included below.

#### ICT Access

We considered ICT access to be a combination of number of ICT devices within the home, and the average estimated use of these devices per week.

- How many phones (e.g., iPhone and Android) does the child’s family have in the home? (*Rated 0 to 5+ devices*).
- How many touch-screen-devices (e.g., iPad, Kindle, and Nintendo DS) does the child’s family have in the home? (*Rated 0 to 5+ devices*).
- How many computers (laptop or desktop) does the child’s family have in the home? (*Rated 0 to 5+ devices*).
- How many video game systems (e.g., PlayStation, Xbox, Wii, and Leapster) does the child’s family have in the home? (*Rated 0 to 5+ devices*).

An additional three questions ask parents to estimate the number of hours each week that the child spends using the devices for any purpose (touchscreen devices and computers are combined). (*Rated 0 to 15+ hours*). Cronbach’s alpha for these seven items was adequate within this sample: α = 0.74.

#### ICT Aptitude

The HEQ uses nine items to measure ICT attitude and aptitude, and our internal reliability analysis suggested that these items had good internal consistency within our sample: α = 0.79. Caregivers were asked to rate their level of agreement with a set of statements asking about their child’s affinity and skill at using ICT. Items were rated from “Strongly Disagree” to “Strongly Agree.”

- My child likes using technology.
- My child easily learns new technology.
- My child has trouble figuring out new technology (reverse coded).
- My child uses technology all the time.
- My child often checks for updates or texts.
- My child is confident about using technology.
- My child wants the latest in technology.
- My child is slow to learn new technology (reverse coded).
- My child is an expert at using technology.

#### E-Learning

Caregivers were asked to estimate the number of hours per week their child spent engaging in E-Learning, which we have operationalised here as engaging in utilizing ICT for educational purposes (such as homework). We have also included the caregiver estimate of time spent watching educational television programs, such as documentaries. Our reliability analysis of these items suggested that the two items were orthogonal to one another, with a nearly null correlation (*r* = −0.03, *p* = 0.341).

- How many hours per week does the child use smartphones, tablets, and computers for homework or school-related activities?
- How many hours per week does the child usually watch educational or informative television programs?

#### Recreational ICT

Caregivers were asked to estimate the number of hours per week their child used ICT for recreational purposes.

- How many hours per week does the child use smartphones, tablets, and computers for surfing the web for fun?
- How many hours per week does the child use smartphones, tablets, and computers for social media (e.g., Facebook, Twitter, and text-messaging)?
- How many hours per week does the child use smartphones, tablets, and computers for playing games?
- How many hours per week does the child use smartphones, tablets, and computers for listening to music/watching movies?
- How many hours per week does the child use smartphones, tablets, and computers for creative or artistic activities (e.g., art and photography)?

Our analysis suggested that this set of questions had reasonable internal consistency for our sample: α = 0.75.

#### Socio-Economic Status (SES)

Socio-economic status was based upon parent-reported total gross family income per year, and highest educational attainment among caregivers. Income was categorized in $5k incremental blocks from $0 to $20k per year, in $10k increments from $20k to $80k, in $20k increments from $80K to 100K, $25K increments to $200k, and $50k increments beyond. Caregiver educational attainment was categorized into four levels: non-completion of high school; completed high school; completion of TAFE certification/Associates degree; and completion of an undergraduate or post-graduate degree.

#### Institutional Approval

Approval for the WIAT-III A&NZ standardization project was obtained from the University of Sydney Ethics Committee (Human Research) and the relevant ethics committees of school authorities in each state and territory where required. As this analysis only involved the use of pre-existing, non-identifiable data and/or publicly available data, the present analysis was exempt from ethical review (DUHREC [REDACTED]). Only participants who completed both the HEQ and the WIAT-III A&NZ were included in this study.

### Data Analysis

All relevant HEQ variables were mapped against the WIAT-III A&NZ academic indices, being consistent in numerical form and appropriate for regression analysis. Before analysis, all variables were examined for univariate and multivariate normality through visual inspection of P–P and Q–Q plots and data met assumptions. Multicollinearity of independent variables was analyzed in each model, with multicollinearity flagged for variables with tolerance values <0.200. Analysis suggested no multicollinearity among IVs. Models one to four present the sequential multiple regression analyses of individual factors of ICT use, broadly grouped into factors related to ICT home access, child ICT aptitude, child experience of learning through technology at home (“E-Learning”), and the relative associations of child recreational ICT use with each of the four academic metrics. Finally, this study considered that the age of the child would likely be confound in the relationship between ICT use and academic performance. Simply, younger participants would likely interact with technology differently than older participants and an older student would be expected to use technology for longer durations, as well as have greater access to different ICT resources. Thus, each model included each prior ICT factor, and adjusted for child age, parental education, and familial income.

#### Sequential Multiple Regression Analysis Plan

To account for the correlated nature of the independent variables in the analysis and the multiple dependent variables, we elected to use multiple regression to analyze our models. Our planned models were designed to test each hypothesis with as parsimonious a model as possible, guided by internal consistency analysis for models one, two, and four, and by apparent relevance to E-learning for model three. Using the *n* = 714 parent records sample, models were built to reflect the hypotheses using the WIAT-III A&NZ academic indices (Reading, Writing, Oral, and Mathematics), home ICT factors, child age, household income, and parental educational attainment. The relative size of the standardized regression coefficient β can be interpreted according to the heuristic of ±0.05 to 0.25 = small, ±0.25 to 0.45 = medium, and ±0.5 or greater = large (Peterson and Brown, 2005). In all models we used sequential regression to quantify the combined effect of ICT variables (*step two*) once household income, parental income, and child age were accounted for, as an *R*^2^ adjusted for multiple IVs. Given the exploratory nature of the models, we have not adjusted the significance of individual predictors for multiple comparisons.

Finally, we performed a *post hoc* exploratory analysis of the final model after excluding the youngest children.

## Results

### Descriptive Statistics

Table 1 presents the descriptive statistics for key variables used in the inferential models. On average, children within the sample spent the most amount of screen-time using a mobile phone or playing video games. Reported hours of use were lowest for creative arts pursuits.

**TABLE 1:** Descriptive statistics.

	*N*	%	Minimum	Maximum	*M*	*SD*	Mean range
Gender (female)	370	51.8					
Child owns a touch screen phone (yes)	335	46.9					
Child has a social media account (yes)	230	32.2					
Highest parent education level							
Did not complete secondary education	108	15.0					
Secondary education	163	22.7					
TAFE certification/associates	237	33.0					
Tertiary education	210	29.2					
Family income			1	16	7.81	3.19	$60k–$69k
Age			4.06	18.31	10.12	3.68	
Reading			58	143	100.82	14.591	
Mathematics			62	154	101.77	14.282	
Written expression			64	146	101.91	14.446	
Oral language			57	149	101.73	14.299	
Video game systems in the home			0	5	2.15	1.553	
Computers in the home			0	5	2.3	1.287	
Touchscreen devices in the home			0	5	2.35	1.576	
Mobile phones in the home			0	5	2.67	1.22	
Hours watching educational TV			0	15+	1.85	1.415	2–4 h
Hours playing video games			0	15+	2.39	1.59	2–4 h
Hours using mobile phones			0	15+	2.37	2.31	4–6 h
Hours using computers or touchscreen devices			0	15+	2.1	1.223	6–8 h
Hours surfing the web for fun			0	15+	1.1	1.473	2–4 h
Hours on social media			0	15+	0.65	1.534	1–2 h
Hours playing games for fun			0	15+	1.66	1.548	2–4 h
Hours listening to music or watching movies			0	15+	1.59	1.795	2–4 h
Hours creating art			0	15+	0.65	0.992	1–2 h
Hours doing homework on ICT devices			0	15+	1.62	1.721	4–6 h
Child likes using technology			1	6	5.65	0.672	
Child easily learns			1	6	5.51	0.823	
Child has trouble with technology			1	6	1.8	1.163	
Child uses technology often			1	6	4.37	1.564	
Child frequently checks for updates or texts			1	6	2.53	1.854	
Child is confident using technology			1	6	5.35	0.964	
Child wants the latest technology			1	6	3.93	1.748	
Child is slow to learn technology			1	6	1.62	1.055	
Child is an expert at using technology			1	6	3.86	1.462	

### Correlations

Table 2 presents the bivariate correlations between each of the ICT variables used in the models, household income, parental educational attainment, and child age. The strength of the association’s ranges from trivial to strong, and the strongest correlation was between child preference and aptitude for ICT use (*r* = 0.652, *p* < 0.001).

**TABLE 2:** Bivariate correlations between ICT variables.

	1	2	3	4	5	6	7	8	9	10	11	12	13	14	15	16	17	18	19	20	21	22	23	24	25	26
(1)	1																									
(2)	0.048																									
(3)	0.003	0.503^†^																								
(4)	0.102^†^	−0.013	−0.089*																							
(5)	0.041	−0.108^†^	−0.142^†^	0.354^†^																						
(6)	0.409^†^	0.138^†^	0.03	0.244^†^	0.061																					
(7)	0.453^†^	0.007	−0.06	0.224^†^	0.188^†^	0.400^†^																				
(8)	0.372^†^	0.236^†^	0.264^†^	0.206^†^	0.06	0.377^†^	0.159^†^																			
(9)	0.159^†^	0.219^†^	0.077*	0.508^†^	0.157^†^	0.298^†^	0.238^†^	0.346^†^																		
(10)	0.546^†^	0.041	−0.017	0.296^†^	0.279^†^	0.335^†^	0.573^†^	0.360^†^	0.340^†^																	
(11)	−0.173^†^	−0.114^†^	−0.113^†^	0.061	0.218^†^	−0.044	0.046	−0.045	0.023	0.013																
(12)	0.117^†^	−0.102^†^	−0.121^†^	0.155^†^	0.178^†^	0.043	0.269^†^	0.018	0.083^†^	0.263^†^	0.220^†^															
(13)	0.505^†^	−0.02	−0.095^†^	0.103^†^	0.126^†^	0.312^†^	0.458^†^	0.219^†^	0.128^†^	0.498^†^	−0.007	0.244^†^														
(14)	0.555^†^	−0.009	−0.049	0.059	−0.031	0.320^†^	0.555^†^	0.168^†^	0.078*	0.377^†^	−0.103^†^	0.187^†^	0.531^†^													
150.	0.535^†^	0.097^†^	0.071	0.089^†^	0.075*	0.292^†^	0.369^†^	0.335^†^	0.178^†^	0.489^†^	−0.042	0.104^†^	0.403^†^	0.405^†^												
(16)	0.241^†^	−0.049	−0.082*	0.238^†^	0.437^†^	0.164^†^	0.333^†^	0.156^†^	0.168^†^	0.514^†^	0.072*	0.300^†^	0.397^†^	0.174^†^	0.206^†^											
(17)	0.463^†^	−0.079*	−0.079*	0.153^†^	0.105^†^	0.291^†^	0.490^†^	0.176^†^	0.177^†^	0.480^†^	0.014	0.273^†^	0.562^†^	0.608^†^	0.427^†^	0.346^†^										
(18)	0.196^†^	−0.147^†^	−0.108^†^	0.153^†^	0.219^†^	0.133^†^	0.225^†^	0.061	0.145^†^	0.298^†^	0.081*	0.082^†^	0.297^†^	0.270^†^	0.376^†^	0.258^†^	0.384^†^									
(19)	0.180^†^	0.02	−0.01	0.110^†^	0.157^†^	0.118^†^	0.183^†^	0.106^†^	0.131^†^	0.235^†^	0.057	0.081^†^	0.152^†^	0.122^†^	0.114^†^	0.226^†^	0.138^†^	0.068*								
(20)	0.123^†^	0.04	0.003	0.098^†^	0.106^†^	0.112^†^	0.160^†^	0.053	0.081^†^	0.196^†^	0.031	0.053	0.094^†^	0.096^†^	0.114^†^	0.168^†^	0.090^†^	0.073*	0.652^†^							
(21)	−0.132^†^	−0.089^†^	−0.128^†^	−0.013	−0.047	−0.044	−0.120^†^	−0.101^†^	−0.073*	−0.129^†^	0.032	0.016	−0.075*	−0.056	−0.111^†^	−0.120^†^	−0.009	−0.055	−0.326^†^	−0.502^†^						
(22)	0.452^†^	−0.069*	−0.138^†^	0.255^†^	0.275^†^	0.320^†^	0.419^†^	0.233^†^	0.225^†^	0.528^†^	0.041	0.237^†^	0.387^†^	0.311^†^	0.317^†^	0.394^†^	0.333^†^	0.227^†^	0.381^†^	0.312^†^	−0.219^†^					
(23)	0.645^†^	−0.03	−0.032	0.073*	0.046	0.371^†^	0.466^†^	0.234^†^	0.108^†^	0.407^†^	−0.048	0.118^†^	0.440^†^	0.573^†^	0.393^†^	0.248^†^	0.415^†^	0.208^†^	0.171^†^	0.121^†^	−0.059	0.459^†^				
(24)	0.295^†^	0.034	0.005	0.095^†^	0.135^†^	0.166^†^	0.222^†^	0.137^†^	0.146^†^	0.272^†^	0.017	0.093^†^	0.172^†^	0.162^†^	0.216^†^	0.232^†^	0.157^†^	0.098^†^	0.539^†^	0.625^†^	−0.465^†^	0.393^†^	0.250^†^			
(25)	0.370^†^	−0.044	−0.095^†^	0.132^†^	0.108^†^	0.155^†^	0.283^†^	0.079*	0.096^†^	0.289^†^	−0.005	0.110^†^	0.276^†^	0.245^†^	0.222^†^	0.227^†^	0.207^†^	0.135^†^	0.367^†^	0.275^†^	−0.197^†^	0.448^†^	0.412^†^	0.395^†^		
(26)	−0.138^†^	−0.082*	−0.130^†^	−0.036	−0.05	−0.092^†^	−0.098^†^	−0.138^†^	−0.056	−0.155^†^	0.002	0.018	−0.061	−0.090^†^	−0.136^†^	−0.130^†^	−0.033	−0.034	−0.371^†^	−0.543^†^	0.617^†^	−0.260^†^	−0.107^†^	−0.516^†^	−0.217^†^	
(27)	0.358^†^	−0.001	−0.023	0.130^†^	0.180^†^	0.222^†^	0.238^†^	0.192^†^	0.136^†^	0.332^†^	0.053	0.054	0.249^†^	0.179^†^	0.251^†^	0.285^†^	0.145^†^	0.122^†^	0.385^†^	0.445^†^	−0.368^†^	0.478^†^	0.358^†^	0.544^†^	0.463^†^	−0.395^†^

(1) Age; (2) income; (3) PED; (4) #Videogames; (5) videogame hours; (6) #Phones; (7) phone hours; (8) #Computers; (9) #Touchscreen; (10) computer hours; (11) educational TV; (12) entertainment TV; (13) surf the web; (14) social media; (15) eLearning; (16) gaming; (17) music or movies; (18) art; (19) likes ICT; (20) easily learns ICT; (21) trouble with ICT; (22) uses ICT often; (23) updates or texts; (24) confident with ICT; (25) wants latest ICT; (26) slow at ICT; (27) expert at ICT. *p < 0.05, ^†^p < 0.01.

### Hypothesis One

The regression coefficients are presented in Table 3. Household income and parental education were significant, positive predictors of academic performance on each index, and parental education was the strongest predictor for all indices. Child age was not significantly associated with any index. For ICT access variables, the number of computers in the home (but not hours spent using the computers) was significantly and positively associated with performance on the mathematics index. Increased weekly hours playing videogames had a small, but significant negative association with written language. As a combination, the addition of ICT variables failed to significantly account for a significant proportion of the variance in any index.

**TABLE 3:** Sequential multiple regression of ICT Access on WIAT-III A&NZ Academic indices.

DV	IV	BETA	*B*	*SE*	*T*	*P*
**WIAT-III A&NZ mathematics**						
	Household income	0.1997	0.858	0.179	4.782	<0.001
	Parental education	0.2537	3.578	0.595	6.018	<0.001
	Child age	0.0116	0.046	0.184	0.248	0.804
	#Videogame systems	0.0467	0.424	0.416	1.019	0.308
	Videogame hours	−0.0252	−0.254	0.432	−0.589	0.575
	#Phones	0.0168	0.195	0.485	0.402	0.688
	Phone hours	−0.0718	−0.441	0.278	−1.588	0.112
	#Computers	0.0999	1.080	0.460	2.347	0.019
	#Touchscreens	−0.0512	−0.467	0.412	−1.135	0.256
	#Touchscreen and computer hours	0.0966	1.130	0.599	1.887	0.059
	Total *R*^2^ = 0.184, *p* < 0.001					
	ICT Δ*R*^2^ = 0.019, *p* = 0.058					
**WIAT-III A&NZ oral language**						
	Household income	0.183	0.807	0.178	4.542	<0.001
	Parental education	0.297	4.284	0.584	7.332	<0.001
	Child age	0.050	0.202	0.178	1.136	0.256
	#Videogame systems	0.017	0.159	0.395	0.403	0.687
	Videogame hours	−0.034	−0.348	0.389	−0.892	0.372
	#Phones	−0.041	−0.488	0.486	−1.004	0.315
	Phone hours	−0.024	−0.150	0.279	−0.536	0.592
	#Computers	0.076	0.838	0.460	1.819	0.069
	#Touchscreens	−0.019	−0.179	0.405	−0.442	0.659
	#Touchscreen and computer hours	−0.015	−0.175	0.584	−0.300	0.764
	*R*^2^ = 0.196, *p* < 0.001					
	ICT Δ*R*^2^ = 0.007, *p* = 0.513					
**WIAT-III A&NZ reading**						
	Household income	0.165	0.739	0.194	3.802	<0.001
	Parental education	0.263	3.852	0.658	5.851	<0.001
	Child age	−0.007	−0.029	0.204	−0.143	0.886
	#Videogame systems	−0.003	−0.031	0.455	−0.068	0.946
	Videogame hours	−0.049	−0.518	0.427	−1.213	0.225
	#Phones	−0.038	−0.456	0.522	−0.873	0.383
	Phone hours	−0.032	−0.206	0.297	−0.694	0.488
	#Computers	0.003	0.039	0.502	0.077	0.939
	#Touchscreens	−0.016	−0.149	0.448	−0.332	0.740
	#Touchscreen and computer hours	0.047	0.571	0.636	0.898	0.369
	*R^2^ = 0.144, p < 0.001*					
	ICT Δ*R*^2^ = 0.005, *p* = 0.667					
**WIAT-III A&NZ written expression**						
	Household income	0.127	0.551	0.186	2.966	0.003
	Parental education	0.265	3.767	0.627	6.008	<0.001
	Child age	0.047	0.187	0.196	0.953	0.341
	#Videogame systems	0.009	0.083	0.416	0.201	0.841
	Videogame hours	−0.079	−0.805	0.398	−2.022	0.043
	#Phones	−0.019	−0.221	0.504	−0.438	0.661
	Phone hours	−0.047	−0.290	0.284	−1.023	0.306
	#Computers	0.069	0.755	0.482	1.568	0.117
	#Touchscreens	−0.001	−0.014	0.420	−0.032	0.974
	#Touchscreen and Computer hours	0.073	0.857	0.607	1.412	0.158
	*R*^2^ = 0.158, *p* < 0.001					
	ICT Δ*R*^2^ = 0.012, *p* = 0.204					

### Hypothesis Two

Household income and parental education were significant, positive predictors of academic performance on each index, and parental education was the strongest predictor for all indices (see Table 4). Child age was not significantly associated with any index. Parental perception of their children as experts in ICT use was positively associated with mathematics ability, and frequently checking for updates or texts was positively associated with written expression. In contrast, parental report of children using often technology was negatively associated with reading, written expression, and oral language ability indices, and children whose parents reported that they were slower to learn technology recorded lower scores on the reading and written expression indices. None of child confidence, affinity for ICT, desire for latest technology, or perception of difficulty using ICT were associated with any index. The prediction of each index was significantly improved by the addition of the ICT aptitude variables, but the effect sizes were minute.

**TABLE 4:** Sequential multiple regression of ICT Aptitude for WIAT-III A&NZ Academic indices.

DV	IV	BETA	*B*	*SE*	*T*	*P*
**WIAT-III A&NZ mathematics**						
	Household income	0.210	0.905	0.176	5.141	<0.001
	Parental education	0.243	3.446	0.591	5.831	<0.001
	Child age	0.038	0.154	0.197	0.784	0.433
	Likes using technology	0.019	0.435	1.118	0.389	0.697
	Easily learns technology	−0.048	−0.842	0.934	−0.901	0.367
	Has trouble with technology	−0.088	−1.152	0.601	−1.916	0.055
	Uses technology often	−0.091	−0.861	0.449	−1.917	0.055
	Updates technology	0.005	0.033	0.369	0.089	0.929
	Confident with technology	−0.028	−0.412	0.824	−0.500	0.617
	Wants latest technology	0.028	0.234	0.374	0.625	0.532
	Slow to learn technology	−0.070	−0.983	0.696	−1.413	0.158
	Expert at using technology	0.111	1.092	0.490	2.228	0.026
	*R*^2^ = 0.182, *p* < 0.001					
	ICT Δ*R*^2^ = 0.03, *p* = 0.004					
**WIAT-III A&NZ oral language**						
	Household income	0.170	0.755	0.175	4.313	<0.001
	Parental education	0.297	4.285	0.583	7.351	<0.001
	Child age	0.063	0.255	0.190	1.343	0.179
	Likes using technology	0.005	0.108	1.107	0.098	0.922
	Easily learns technology	0.004	0.075	0.917	0.082	0.935
	Has trouble with technology	−0.062	−0.829	0.593	−1.398	0.162
	Uses technology often	−0.105	−1.011	0.442	−2.287	0.022
	Updates technology	−0.057	−0.450	0.369	−1.220	0.223
	Confident with technology	0.043	0.671	0.786	0.854	0.393
	Wants latest technology	0.056	0.474	0.368	1.286	0.198
	Slow to learn technology	−0.054	−0.787	0.669	−1.177	0.239
	Expert at using technology	0.026	0.267	0.472	0.565	0.572
	*R*^2^ = 0.192, *p* < 0.001					
	ICT Δ*R*^2^ = 0.024, *p* = 0.011					
**WIAT-III A&NZ reading**						
	Household income	0.158	0.710	0.189	3.758	<0.001
	Parental education	0.247	3.625	0.653	5.548	<0.001
	Child age	−0.027	−0.110	0.216	−0.511	0.610
	Likes using technology	−0.025	−0.613	1.231	−0.498	0.619
	Easily learns technology	−0.017	−0.294	1.014	−0.290	0.772
	Has trouble with technology	−0.044	−0.597	0.663	−0.900	0.368
	Uses technology often	−0.108	−1.064	0.488	−2.179	0.029
	Updates technology	−0.003	−0.022	0.394	−0.056	0.955
	Confident with technology	0.036	0.585	0.868	0.674	0.500
	Wants latest technology	0.063	0.555	0.412	1.348	0.178
	Slow to learn technology	−0.118	−1.721	0.750	−2.295	0.022
	Expert at using technology	0.036	0.370	0.530	0.698	0.485
	*R*^2^ = 0.147, *p* < 0.001					
	ICT Δ*R*^2^ = 0.031, *p* = 0.004					
**WIAT-III A&NZ written expression**						
	Household income	0.140	0.609	0.181	3.358	0.001
	Parental education	0.258	3.672	0.617	5.955	<0.001
	Child age	0.017	0.069	0.204	0.336	0.737
	Likes using technology	−0.044	−1.014	1.153	−0.880	0.379
	Easily learns technology	−0.012	−0.197	0.952	−0.207	0.836
	Has trouble with technology	−0.054	−0.717	0.625	−1.146	0.252
	Uses technology often	−0.116	−1.105	0.461	−2.399	0.016
	Updates technology	0.115	0.884	0.380	2.327	0.020
	Confident with technology	0.039	0.613	0.855	0.717	0.474
	Wants latest technology	0.022	0.191	0.394	0.484	0.629
	Slow to learn technology	−0.107	−1.517	0.712	−2.132	0.033
	Expert at using technology	0.017	0.171	0.505	0.339	0.735
	*R*^2^ = 0.153, *p* < 0.001					
	ICT Δ*R*^2^ = 0.033, *p* = 0.002					

### Hypothesis Three

Household income and parental education were significant, positive predictors of academic performance on each index, and parental education was the strongest predictor for all indices. Child age was not significantly associated with any index. Time spent watching educational TV was negatively associated with mathematics, oral language, and written expression, while time spent doing homework with ICT was positively associated with mathematics (Table 5). Prediction of all indices except for reading were significantly improved by the addition of the E-learning variables, but the effect size was trivial in each case.

**TABLE 5:** Sequential multiple regression of eLearning for WIAT-III A&NZ Academic indices.

DV	IV	BETA	*B*	*SE*	*T*	*P*
**WIAT-III A&NZ mathematics**						
	Household income	0.197	0.842	0.174	4.837	<0.001
	Parental education	0.259	3.664	0.571	6.418	<0.001
	Child age	−0.001	−0.001	0.167	−0.007	0.994
	Educational TV	−0.117	−1.150	0.373	−3.085	0.002
	Homework using ICT	0.089	0.774	0.352	2.201	0.028
	*R*^2^ = 0.184, *p* < 0.001					
	ICT Δ*R*^2^ = 0.017, *p* = 0.001					
**WIAT-III A&NZ oral language**						
	Household income	0.177	0.780	0.172	4.533	<0.001
	Parental education	0.311	4.485	0.563	7.971	<0.001
	Child age	0.030	0.118	0.161	0.735	0.462
	Educational TV	−0.097	−0.978	0.349	−2.797	0.005
	Homework using ICT	−0.009	−0.084	0.356	−0.235	0.814
	*R*^2^ = 0.196, *p* < 0.001					
	ICT Δ*R*^2^ = 0.009, *p* = 0.02					
**WIAT-III A&NZ reading**						
	Household income	0.154	0.686	0.189	3.633	<0.001
	Parental education	0.268	3.922	0.639	6.142	<0.001
	Child age	−0.021	−0.082	0.180	−0.456	0.648
	Educational TV	−0.073	−0.758	0.419	−1.808	0.071
	Homework using ICT	−0.011	−0.103	0.383	−0.267	0.789
	*R*^2^ = 0.144, *p* < 0.001					
	ICT Δ*R*^2^ = 0.005, *p* = 0.17					
**WIAT-III A&NZ written expression**						
	Household income	0.125	0.538	0.181	2.975	0.003
	Parental education	0.277	3.942	0.601	6.560	0.000
	Child age	0.027	0.107	0.175	0.610	0.542
	Educational TV	−0.100	−0.998	0.372	−2.679	0.007
	Homework using ICT	0.073	0.637	0.362	1.759	0.079
	*R*^2^ = 0.154, *p* < 0.001.					
	ICT Δ*R*^2^ = 0.01, *p* = 0.007					

### Hypothesis Four

Household income and parental education were significant, positive predictors of academic performance on each index, and parental education was the strongest predictor for all indices. Child age was a significant positive predictor all indices except for reading ability. Recreational ICT use factors were not significantly associated with any academic performance metric, and the inclusion of ICT recreation variables did not significantly improve prediction of any academic indices (Table 6).

**TABLE 6:** Sequential multiple regression of recreational ICT use for WIAT-III A&NZ Academic indices.

DV	IV	BETA	*B*	*SE*	*T*	*P*
**WIAT-III A&NZ mathematics**						
	Household income	0.204	0.869	0.176	4.927	<0.001
	Parental education	0.273	3.866	0.575	6.724	<0.001
	Child age	0.114	0.452	0.183	2.477	0.013
	Surfing the web	0.030	0.300	0.450	0.666	0.506
	Social media	−0.064	−0.592	0.441	−1.344	0.179
	Games	0.032	0.305	0.368	0.828	0.408
	Movies/Music	−0.068	−0.535	0.380	−1.410	0.159
	Creative/Arts	−0.026	−0.404	0.576	−0.702	0.483
	*R*^2^ = 184, *p* < 0.001					
	ICT Δ*R*^2^ = 0.01, *p* = 0.11					
**WIAT-III A&NZ oral language**						
	Household income	0.185	0.813	0.173	4.695	<0.001
	Parental education	0.317	4.578	0.565	8.100	<0.001
	Child age	0.095	0.383	0.173	2.206	0.027
	Surfing the web	0.001	0.010	0.456	0.021	0.983
	Social media	−0.092	−0.871	0.446	−1.952	0.051
	Games	0.030	0.285	0.371	0.769	0.442
	Movies/Music	−0.033	−0.270	0.386	−0.699	0.485
	Creative/Arts	0.030	0.454	0.576	0.788	0.430
	*R*^2^ = 196, *p* < 0.001					
	ICT Δ*R*^2^ = 0.01, *p* = 0.16					
**WIAT-III A&NZ reading**						
	Household income	0.150	0.669	0.190	3.524	<0.001
	Parental education	0.272	3.991	0.640	6.238	<0.001
	Child age	0.033	0.137	0.199	0.688	0.492
	Surfing the web	0.033	0.346	0.483	0.716	0.474
	Social media	−0.058	−0.558	0.474	−1.179	0.239
	Games	−0.013	−0.132	0.396	−0.333	0.739
	Movies/music	−0.053	−0.433	0.419	−1.033	0.302
	Creative/arts	−0.020	−0.314	0.624	−0.504	0.615
	*R*^2^ = 144, *p* < 0.001					
	ICT Δ*R*^2^ = 0.007, *p* = 0.323					
**WIAT-III A&NZ written expression**						
	Household income	0.134	0.576	0.183	3.146	0.002
	Parental education	0.284	4.046	0.605	6.687	<0.001
	Child age	0.099	0.396	0.191	2.072	0.039
	Surfing the web	−0.026	−0.259	0.466	−0.556	0.578
	Social media	0.043	0.397	0.455	0.872	0.383
	Games	−0.044	−0.418	0.378	−1.108	0.268
	Movies/music	−0.039	−0.307	0.392	−0.782	0.434
	Creative/arts	−0.007	−0.116	0.594	−0.195	0.845
	*R*^2^ = 15, *p* < 0.001					
	ICT Δ*R*^2^ = 0.005, *p* = 0.484					

### Combined Model – All IVs

Model five combines each ICT access factor from the prior models and tests the model improvement over socioeconomic factors and child age (Table 7). We observed that household income and parental education were positively associated with each index, and that parental education was the strongest predictor even after the inclusion of ICT factors. Number of household computers was positively associated with mathematics and oral language, while time spent watching educational TV was negatively associated with each index except for reading. Time spent on social media was negatively associated with oral language. The regression pathways in model five accounted for between 14.5 and 19.7% of the variance in the WIAT-III A&NZ academic indices, accounting for a moderate portion of variance in each. Crucially, the inclusion of ICT access factors accounted for trivial proportions of variance for the mathematics and oral language indices and did not significantly increase the adjusted *R*^2^ for the reading or written expression indices.

**TABLE 7:** Combined model of ICT variables for WIAT-III A&NZ academic indices.

DV	IV	BETA	*B*	*SE.*	*T*	*P*
**WIAT-III A&NZ mathematics**						
	Household income	0.175	0.742	0.181	4.093	<0.001
	Parental education	0.245	3.455	0.593	5.825	<0.001
	Age	−0.005	−0.018	0.208	−0.084	0.933
	#Videogame systems	0.031	0.280	0.410	0.684	0.494
	Hours playing videogames	−0.018	−0.160	0.376	−0.426	0.670
	#Mobile phones	0.018	0.222	0.483	0.459	0.646
	Hours spent using mobile phones	−0.020	−0.117	0.307	−0.383	0.702
	#Touchscreen devices	−0.032	−0.293	0.411	−0.714	0.475
	Hours spent using computers or touchscreen devices	0.079	0.918	0.630	1.458	0.145
	#Computers	0.090	0.978	0.463	2.114	0.035
	Hours surfing the web	0.016	0.160	0.452	0.353	0.724
	Hours using social Media	−0.058	−0.542	0.475	−1.143	0.253
	Hours playing games online	0.025	0.245	0.423	0.579	0.563
	Hours Listening to music/watching movies	−0.085	−0.667	0.382	−1.747	0.081
	Hours creative pursuits	−0.022	−0.331	0.588	−0.563	0.573
	Educational TV	−0.118	−1.169	0.385	−3.038	0.002
	eLearning/homework	0.085	0.732	0.378	1.937	0.053
	*R*^2^ = 184, *p* < 0.001					
	ICT Δ*R*^2^ = 0.042, *p* = 0.001					
**WIAT-III A&NZ oral language**						
	Household income	0.182	0.798	0.180	4.438	<0.001
	Parental education	0.291	4.184	0.584	7.160	<0.001
	Age	0.072	0.287	0.205	1.400	0.161
	#Videogame systems	−0.012	−0.106	0.396	−0.268	0.789
	Playing games on the internet	0.007	0.067	0.385	0.173	0.863
	#Mobile phones	−0.036	−0.427	0.485	−0.880	0.379
	Hours spent using mobile phones	0.038	0.232	0.310	0.750	0.453
	#Touchscreen devices	−0.012	−0.115	0.410	−0.280	0.780
	Hours spent using computers or touchscreen devices	−0.045	−0.543	0.631	−0.861	0.389
	#Computers	0.082	0.914	0.464	1.969	0.049
	Hours surfing the web	0.001	0.013	0.463	0.027	0.978
	Hours using social Media	−0.107	−1.000	0.484	−2.065	0.039
	Hours playing games online	0.043	0.422	0.430	0.981	0.327
	Hours Listening to music/watching movies	−0.020	−0.168	0.393	−0.427	0.670
	Hours creative pursuits	0.050	0.781	0.594	1.316	0.188
	Educational TV	−0.112	−1.131	0.362	−3.125	0.002
	eLearning/homework	−0.015	−0.127	0.385	−0.329	0.742
	*R*^2^ = 197, *p* < 0.001					
	ICT Δ*R*^2^ = 0.025, *p* = 0.007					
**WIAT-III A&NZ reading**						
	Household income	0.155	0.683	0.198	3.455	0.001
	Parental education	0.261	3.821	0.668	5.722	<0.001
	Age	0.008	0.035	0.230	0.152	0.879
	#Videogame systems	−0.026	−0.248	0.464	−0.533	0.594
	Hours playing video games	0.020	0.182	0.410	0.443	0.658
	#Mobile phones	−0.036	−0.429	0.524	−0.818	0.413
	Hours spent using mobile phones	0.015	0.099	0.333	0.299	0.765
	#Touchscreen devices	−0.003	−0.022	0.455	−0.048	0.962
	Hours spent using computers or touchscreen devices	0.045	0.545	0.687	0.793	0.428
	#Computers	0.002	0.026	0.511	0.052	0.959
	Hours surfing the web	0.030	0.305	0.493	0.619	0.536
	Hours using social media	−0.064	−0.629	0.519	−1.213	0.225
	Hours playing games online	−0.029	−0.271	0.462	−0.586	0.558
	Hours listening to music/watching movies	−0.054	−0.429	0.428	−1.003	0.316
	Hours creative pursuits	−0.011	−0.179	0.649	−0.275	0.783
	Educational TV	−0.075	−0.781	0.434	−1.800	0.073
	eLearning/homework	−0.001	−0.010	0.421	−0.023	0.981
	*R*^2^ = 145, *p* < 0.001					
	ICT Δ*R*^2^ = 0.016, *p* = 0.522					
**WIAT-III A&NZ written expression**						
	Household income	0.111	0.474	0.188	2.518	0.012
	Parental education	0.258	3.670	0.630	5.829	<0.000
	Age	0.012	0.050	0.224	0.223	0.824
	#Videogame systems	0.003	0.023	0.419	0.055	0.956
	Hours playing video games	−0.049	−0.438	0.393	−1.116	0.264
	#Mobile phones	−0.019	−0.218	0.503	−0.434	0.664
	Hours spent using mobile phones	−0.049	−0.295	0.316	−0.933	0.351
	#Touchscreen devices	0.007	0.061	0.424	0.143	0.886
	Hours spent using computers or touchscreen devices	0.106	1.240	0.656	1.889	0.059
	#Computers	0.068	0.741	0.488	1.520	0.129
	Hours surfing the web	−0.036	−0.352	0.470	−0.749	0.454
	Hours using social media	0.060	0.548	0.491	1.116	0.265
	Hours playing games online	−0.042	−0.389	0.436	−0.891	0.373
	Hours Listening to music/watching movies	−0.051	−0.411	0.398	−1.033	0.302
	Hours creative pursuits	−0.003	−0.048	0.611	−0.078	0.938
	Educational TV	−0.089	−0.893	0.385	−2.317	0.021
	eLearning/homework	0.056	0.487	0.392	1.243	0.214
	*R*^2^ = 151, *p* < 0.001					
	ICT Δ*R*^2^ = 0.028, *p* = 0.052					

### Post hoc Exploration of Social Media and Older Children

Our analyses revealed that unlike all other variables used, social media use was reported as almost entirely absent in children younger than 10 years. To explore whether this heterogeneity impacted upon the analysis, we re-ran the final model excluding all children younger than ten. For mathematics, the regression was significant at step one but did not improve after the inclusion of ICT variables; *R*^2^ = 0.261, *F*(3,314) = 38.367, *p* < 0.001, Δ*R*^2^ = 0.02, *p* = 0.602. The number of household computers and hours spent watching educational TV were no longer predictive of mathematics performance, but time spent engaging in eLearning was positively associated with the DV; β = 0.118, *t* = 1.997, *p* = 0.047. For oral language, the regression was significant at step one but did not improve after the inclusion of ICT variables; *R*^2^ = 0.26, *F*(3,314) = 38.31, *p* < 0.001, Δ*R*^2^ = 0.02, *p* = 0.714. The number of household computers was no longer associated with oral language, but the negative association of social media use marginally strengthened; β = −0.158, *t* = −2.089 *p* = 0.038. For reading the regression model was significant at step one but did not improve after the inclusion of ICT variables; *R*^2^ = 0.185, *F*(3,314) = 24, *p* < 0.001, Δ*R*^2^ = 0.01, *p* = 0.975, and no ICT variables were associated with reading ability. Finally, for written expression the regression model was significant at step one but did not improve after the inclusion of ICT variables; *R*^2^ = 0.237, *F*(3,314) = 33.876, *p* < 0.001, Δ*R*^2^ = 0.034, *p* = 0.455. The prior negative association of educational TV and written expression did not persist in this analysis, but we observed a positive association of time spent engaged in social media and written expression; β = 0.159, *t* = 2.099, *p* = 0.037.

## Discussion

Our study set out to determine the impact of home ICT factors on student’s academic performance, including their access to ICT, their aptitude with ICT, and the amount of time spent in both intentional e-learning and recreational ICT use. This research extends on Skryabin et al. (2015)’s work, which examined the academic enhancement effects of ICT use for both e-learning and entertainment by performing sequential regression modeling of the home ICT factors, adjusting for child age and family socioeconomic status. The five analytical models designed in this research allow for a nuanced understanding of the complex interaction between home technology and achievement in reading, writing, mathematical and oral abilities. Importantly, our inclusion of touchscreen devices, such as tablets, provides an additional avenue of exploration in a rapidly changing landscape of home ICT use.

### ICT Access in the Home

We observed inconsistent support for hypothesis 1, with little evidence to suggest any substantial associations between home ICT access and academic indices once the influence of SES and child age were controlled. We observed that the presence of computers in the home and video game systems had almost inverse associations with academic performance, though both the relative size and statistical significance of the associations were in favor of computers. This result represents a clear delineation between devices intended purely for entertainment, and those devices with functions that extend beyond recreation and are frequently utilized for work or educational purposes (see section “Model Three”). We observed few associations that specifically related to hours spent using ICT devices – weekly time spent playing videogames was the sole significant association, indicating a weakly negative relationship with written expression. The lack of significant associations for the household presence of mobile phones and other touchscreen devices and any academic metric serves to allay a common childrearing fear that household ICT devices must enact a negative force on academics. Strikingly, when we included specific types of ICT use (such as that required for educational purposes) we observed that the use of older technologies, such as educational television, were almost solely responsible for any observed negative associations with academic performance. Further, upon full adjustment for the presence of other ICT access factors, child age, and socioeconomic status we observe that the presence of ICT devices was almost entirely unrelated to academic performance or, in the case of number of household computers, weakly positively associated with oral language and mathematical ability. Importantly, our ability to apportion the model contributions of household income, parental education, and the number of household ICT devices allows us to suggest that there is some advantage to having more ICT devices in the household, beyond having the household resources to afford them. This is further established by our models demonstrating that parental education level is consistently the strongest predictor of academic performance, and our correlational observation that the number of devices in a household is more strongly related to parental education level rather than income.

Our models support the finding of Carrasco and Torrecilla (2012), Casey et al. (2012), and Skryabin et al. (2015) and allows some elucidation on earlier inconsistencies in the broader body of research. For example, we observe that children who live in a more technologically rich environment can perform better across most aspects of academic performance we measured. Previous research has routinely found robust correlations between ICT access and mathematical ability but less so for correlations between home ICT access and other academic domains (Carrasco and Torrecilla, 2012; Skryabin et al., 2015). However, our final model found few associations between ICT access in general, and none of the observed correlations could be described as robust. Given the data set’s comparative recency, these results could be generalized as evidence of the growing cultural shift toward technology acceptance (Plowman et al., 2010) and Australia’s increasing technological saturation in educational settings (Australian Bureau of Statistics, 2018). Repeating this methodological approach on even more recent data sets may provide additional confirmation of these results, particularly in the context of the global Coronavirus pandemic which has seen home-schooling enforced world-wide, resulting in even greater reliance on technology for learning.

### Child ICT Aptitude and Preference

Our analysis of the relationship between ICT aptitude factors and academic performance provided little support for our hypothesis that increased ICT aptitude would positively predict academic performance. Our analysis showed that parent perception of their child as “an expert at using ICT” was positively associated with their mathematics performance but no other index, while parental reports of ICT use frequency were positively associated with each of the other indices. Increased mathematical prowess has been routinely associated with ICT access and preference in children (Carrasco and Torrecilla, 2012; Casey et al., 2012; Skryabin et al., 2015), but we note here that our analysis suggests that child self-confidence with ICT use failed to predict academic performance of any kind, and preference was similarly uninvolved. For parents and carriers, we interpret this finding as an indication that the skills required for ICT use may be academically beneficial even if the child is not particularly enjoying using the ICT, or even when the use is challenging. As with *Model One*, we propose the increased reliance upon ICT education during the Coronavirus pandemic will serve to increase child (and parent) ICT use skills, even if preference and confidence remain constant, and may serve to drive further academic benefit in other academic domains (Dimosthenous et al., 2020; Lehrl et al., 2020).

### eLearning and Home Education Utilizing ICT

When considering the influence of formal eLearning on academic outcomes while controlling for the more passive experience of consumption of educational/informative TV material, results from *model three* provide partial support for the relationship described by our third hypothesis. We hypothesized that a child’s engagement in formal e-learning using home ICT would be significantly associated with improved academic performance. However, our analyses found only negative associations between hours of educational TV watching and each academic index except for reading, and one very weak, positive association between homework ICT use and mathematics. Interestingly, after full multivariate adjustment in *model five*, we observe that the while the negative association of educational TV with academic outcomes remains, the positive association with homework does not. Consistent with prevailing models of learning, we again note the distinction between active participation and passive consumption of materials. Chi (2009) explains the hierarchical benefits on educational outcomes of ‘interactive’ learning (e.g., navigating an educational app) over ‘constructive’ learning (i.e., producing something new from the learning content, such as a coherent paraphrased statement), and ‘constructive’ learning over ‘active’ learning (i.e., doing something, such as searching and pointing); all of which are more effective strategies than passive consumption of learning material (e.g., watching a video). Within the current study, even when that material is purported to be educational, the negative effect on academic outcomes is unique to passively consumed material (i.e., educational TV), rather than e-learning activities, likely because these have the potential to be more interactive, constructive, and/or active.

### Recreational ICT Use and Academic Performance

Our investigation of the impact of student frequency in recreational use of home ICT on academic performance partially supported our fourth hypothesis. The regression coefficients of *model four* suggest that recreational ICT was almost universally unrelated to academic performance except for a single negative association between the use of social media and oral language, which only emerged after adjustment in the full *model five.* While results of other research studies have also identified links between recreational ICT and academic outcomes (Bavelier et al., 2010; Comi et al., 2017), the basis for this hypothesis was the qualitative research of Furlong and Davies (2012) who theorized that recreational ICT use fosters incidental learning. As mentioned above, we identified the surprising result that social media was negatively associated with oral language, which appears inconsistent with Furlong and Davies’ incidental learning framework. Incidental learning is fostered in informal learning environments that provide feedback, social networking, and sharing of resources; each of which is applicable to social media use. Our findings of a negative association between the two constructs may be explained by the disparity in casual language employed on social media and the more formal language required for assessment using tools such as the WIAT-III. though this is admittedly speculative. Finally, our *post hoc* analyses excluding children younger than ten suggested positive associations of both eLearning time and social media use for mathematics and written expression. Our subsequent analyses revealed no moderating effect of age for social media use but a significant interaction between age, eLearning, and mathematics whereby only children younger than 10 years reported negative associations between the variables. Why such an association is observed solely the youngest participants and exclusively for mathematics is not entirely clear; though one explanation is that opportunities for positive informal learning of mathematics is rarer than other domains and subsequent to wider variability (Ramani et al., 2015). Further, mathematics and performance are uniquely associated with anxiety and defeatism, which can be detected in primary-school aged children, predict longitudinal mathematics performance, and is strongly influenced by parent attitudes (Casad et al., 2015). As such, it is possible that the increased influence of parents over child learning in the younger years contributes to this age-effect of eLearning that diminishes with additional exposure as children age.

#### Overarching Findings

Our general support for the hypotheses tested suggest that most home ICT factors, including access, aptitude, and affinity for use of ICT are largely unrelated to child academic performance. We emphasize that our models suggest that ICT use, aptitude, and access appear to make relatively small contributions to academic performance after adjusting socioeconomic status, even in combination. Crucially, we propose that academic concerns due to ICT factors are largely unfounded and suggest that the negative associations identified are better characterized by passivity rather than the medium of ICT. If this is true, then we would expect to see that the effect increases increase with the child’s age, as they move from simple interactions such as games and touch devices to more advanced ICT, such as mobile phone hosted social media and web surfing on a computer.

#### Limitations and Recommendations

Our primary limitation is acknowledging that our data is cross-sectional, and while theoretical directionality can be suggested, we are unable to explore causal effects. For example, we cannot directly address whether ICT aptitude is the product of cognitive capacity, which in turn is closely related to academic performance. Further, we are unable to state definitively whether ICT aptitude contributes to academic performance, or whether academic performance in some way necessitates or encourages continued use and experience with ICT. However, while causation and directionality cannot be confirmed, these results still encourage the development of ICT skills in students, with the aim to improve general learning skills, and longitudinal data or an intervention study may account for these variables in future research.

Another major limitation of this research is the anticipated overlap between the independent variables of these five models. While we were able to rule out factorial multicollinearity within models, and while each model measures unique elements of home ICT use and access, there are likely to be interactions between the measured variables which may alter the reported academic enhancement effect. For example, it is reasonable to assume that a child’s access to technology will determine the use of recreational ICT, and their aptitude through repeated use. While our fifth model accounts for the overlap between independent factors, we did not pursue the potential interaction effects that seem plausible given our analyses, such as the likelihood that the associations between ICT use and academic will differ between children of different ages. Given the plethora of factors identified in this paper that may prove to be moderated by age, we propose to explicitly model these in a future analysis.

Finally, a consideration of our dependent variables would suggest that while the WIAT-III A&NZ is a useful measure of academic performance, it does not capture the cognitive processes that drive academic outcomes. In this study, the WIAT-III A&NZ fulfilled the purposes of measuring academic indices and providing a standardized measure that could be used as a comparison across other cultural populations or as part of a longitudinal study in future research. Future research would also benefit from including a measure of intelligence such as the Wechsler Intelligence Scale for Children (Wechsler, 2016) to better understand the cognitive processes and strengths of this home technology act. As with the potential moderating effect of child age, we call for more sophisticated models that include cognitive process measurements.

## Conclusion

These findings highlight the importance of ICT literacy development in students from a young age, given their constant exposure to digital information and a climate of moral panic about screen time in the home. Our study provides some measure of reassurance to parents and caregivers that home ICT use and education via ICT are not only acceptable in terms of academic outcomes, but likely have some unforeseen advantages that appear to outweigh potential disadvantages. This study provides evidence in support of recent educational programs of the Australian government such as the ICT literacy measure in the NAPLAN, and the addition of ICT as a general life capability in the National Curriculum (Thomson, 2015; Australian Curriculum, Assessment and Reporting Authority [ACARA], 2019). However, it may be more important for the future development of educational programs and pedagogy to consider the indirect effects of students’ home ICT use on their learning.

## Data Availability Statement

The data generated for this study is subject to the following licenses/restrictions: The dataset is part of the Pearson Clinical WIAT-III Australia and New Zealand normative sample, and can be sought from Pearson Clinical. Requests to access these datasets should be directed to CW, chris.wilson@pearson.com and NJ, nicki.joshua@pearson.com.

## Ethics Statement

The studies involving human participants were reviewed and approved by Deakin University REC 2018-215. Written informed consent to participate in this study was provided by the participants’ legal guardian/next of kin.

## Author Contributions

DS and MT performed the original statistical analyses and data cleaning. TH, CW, and NJ assisted with the interpretation of results and informed on the application of the output for current educational trends. LB oversaw the development of the original research design and methodology. All authors contributed to the writing and editing of the final manuscript.

## Conflict of Interest

CW and NJ were employed by Pearson Clinical Assessment. The remaining authors declare that the research was conducted in the absence of any commercial or financial relationships that could be construed as a potential conflict of interest.
